# Sardinian Law of Lunacy

**Published:** 1857-10-01

**Authors:** 


### SARDINIAN LAW OF LUNACY.

The following bill for the care and treatment of the insane was introduced into the Sardinian Parliament in 1849, by Dr. Bertini, one of the deputies:—

### Chapter I.—*Of Institutions for the Recovery and Relief of the Insane.*

Art. 1. All insane persons by whom the order and security of the public or of their family are compromised, and those whom it is necessary to treat away from their homes, shall be placed in establishments specially set apart for them.

Art. 2. These establishments are either public or private.

Art. 3. The public establishments shall be placed under the control of the public authorities, and their maintenance paid for out of the funds of the administrative divisions and of the communes.

Art. 4. Private establishments, whether conducted by a private individual or by a society, and expressly destined for the reception of one or more insane persons, shall be under the surveillance of the public authorities.

Art. 5. In order to legalize the establishment of a private asylum, the special authorization of the Minister of the Interior shall be obtained.

Art. 6. No insane person shall be detained, except provisionally, in any prison.

Art. 7. In communes where an hospital or hospice exists, an appropriate place shall be set apart in such charitable institutions for the care and relief of the insane, provided that accommodation cannot be obtained for them in a special institution.

In those communes which do not possess an hospital or hospice, a proper place shall be provided for the purpose.

### Chapter II.—*Of the Authorities empowered to Permit or to Order the Admission of the Insane into Asylums.*

Art. 8. The general intendent of the administrative division shall be empowered to licence or to order the admission of the insane into the public or private establishments within the realm.

Art. 9. The Minister of the Interior, with the previous concurrence of the Foreign Minister, may permit the removal of insane persons to foreign asylums.

### Chapter III.—*Of the Conditions requisite for the Admission of the Insane into Public or Private Establishments.*

Art. 10. For the admission of an insane person into a public or private asylum, when the demand has been made by the relatives or guardians, or by other person interested, the following documents are required:—

*a.* A certificate of the birth of the patient, or other document to show the name, surname, and age of the same.

*b.* An affidavit sworn to by two persons before the judge of requests of the district in which the patient resides, of the insane acts committed by him.

*c.* A declaration, sworn to by a physician or surgeon, of the mental state of the patient.

Art. 11. In the case of insane persons, proposed to be removed to foreign asylums, the consent of the family shall likewise be necessary.

Art. 12. The declaration set forth in Art. 10, sect. *c*, shall be void, if made by the physician of the establishment into which the patient is to be received.

This declaration, and also the attestation referred to in Art. 10, sect. *b*, shall be of no effect after the lapse of fifteen days from the date at which they were made.

Art. 13. The demand for admission, accompanied by the documents prescribed in the preceding articles, shall be presented to the intendent of the province by the persons mentioned in Art. 10, or by the syndic of the commune in which the patient is found.

Art. 14. The patient shall not be admissible into the establishment after the lapse of more than ten days from the date of the order of admission and of the presentation. However, if the patient do not reside near the establishment, this time shall be extended in the proportion of one day for every thirty miles of distance between his abode and the asylum.

Chapter IV.—*Of the Rules regulating the Stay of the Insane in Public Establishments, and of the Conditions for their Release.*

Art. 15. It shall be the duty of the commission, as provided for hereafter by Art. 24, to transmit every fifteen days to the general intendent of the division, through the intendent of the province in which the asylum is situate, a report of the physical and mental condition of the patients, and also of the changes by admissions and discharges during the same period.

Art. 16. The aforesaid commission shall transmit every three months to the Minister of the Interior, an exact report on the physical and mental state of all the inmates of the asylum.

Art. 17. The discharge of patients from the asylum shall be authorized under the following circumstances:—

*a.* When restored to mental health.

*b.* When affected only with acute delirium.

*c.* When a demand is made by the patient's family, and the chief physician can certify that, although uncured, he is not dangerous, and readily manageable in his own family.

Art. 18. In all cases the discharge shall be always sanctioned by the intendent-general of the division, and shall moreover be officially intimated and registered.

Art. 19. The application for the discharge of an uncured patient shall be presented to the intendent-general of the division in which the asylum is situate, who, by the sanction and advice of the chief physician of the asylum, shall order the discharge.

Art. 20. Any patient who has been discharged from an asylum, under the provisions of the preceding articles, may be re-admitted within three months from the date of the discharge, on the production of a medical certificate to be submitted to the syndic, testifying to the continuation of the mental infirmity, and to the improbability of the patient continuing to reside with his family without danger to himself or others.

Art. 21. It shall be incumbent on all persons who shall place an insane person in a foreign asylum, to present every thirty days to the Minister of the Interior a precise report of the physical and mental condition of the patient, prepared by the physician of the asylum.

Art. 22. It shall be in the power of the Minister of the Interior, by previous concert with his colleague for foreign affairs, to cause any patient confined in a foreign asylum to return to his own country, provided that this can be done without injury to the patient, and that he can be readily provided for in his own family, and is in the possession of sufficient pecuniary means for his maintenance.

Chapter V.—*Of the Funds for the Support of Public Establishments.*

Art. 23. The costs for the maintenance of public asylums and for the removal of indigent patients shall be defrayed out of the revenues of the administrative division and of the communes, according to an assessment ordered in the manner set forth in the statutes.

Chapter VI.—*Of the Government of Public and of the Surveillance of Private Asylums.*

Art. 24. The King, with the advice of the Minister of the Interior, shall nominate for each public asylum a commission, composed of four members, in addition to the physician-in-chief, who shall be a member *ex-officio*.

Art. 25. The Minister of the Interior, the intendants-general of divisions, the intendants of provinces, and the sanitary boards, shall exercise a surveillance over the private establishments existing in their respective districts.

Art. 26. A special regulation, approved by royal decree, shall determine on the manner of internal administration of public asylums, the staff of paid officers, their respective duties, religious, sanitary, and economical; and in the case of private asylums, all the conditions requisite for admission into them, and for their internal discipline.

---

M. Bonacossa proposed the following amendments:—

1. That public asylums should be wholly or partially supported by the State, and their expenses not thrown entirely upon the divisions and communes, as contemplated by Art. 3.

2. That, as patients are often kept in confinement in their own homes or in the houses of private persons to their detriment, it shall be made imperative on all persons retaining an insane person in their house to report the fact to the syndic of the commune, or to the intendant of the province.

3. That to the assigned conditions for discharge, another should be added—for those patients in whom no sign of insanity has manifested itself after a certain residence.

4. That a temporary power shall be given to the administrative commission (Art. 24) to represent the insane person in his affairs.

5. Lastly, that the cost of maintenance of indigent insane shall be paid out of the provincial treasury, and not be charged to the communes, except as portions of the province; and that the cost shall be regulated by the number of patients belonging to each division respectively.

